# Diatom-Derived Polyunsaturated Aldehydes Are Unlikely to Influence the Microbiota Composition of Laboratory-Cultured Diatoms

**DOI:** 10.3390/life10030029

**Published:** 2020-03-24

**Authors:** Chloe L. Eastabrook, Paul Whitworth, Georgina Robinson, Gary S. Caldwell

**Affiliations:** 1 School of Natural and Environmental Sciences, Newcastle University, Newcastle upon Tyne NE1 7RU, UK; c.l.eastabrook1@ncl.ac.uk (C.L.E.); p.whitworth@ncl.ac.uk (P.W.); 2 Scottish Association for Marine Science, Scottish Marine Institute, Oban PA37 1QA, UK; Georgina.Robinson@sams.ac.uk

**Keywords:** bacterioplankton interactions, *Skeletonema marinoi*, polyunsaturated aldehydes, nitrogen alterations, algae microbiome

## Abstract

Diatom-derived oxylipins, including polyunsaturated aldehydes (PUA), are considered to have infochemical, allelochemical and bacteriostatic properties, with plausible roles as grazing deterrents and regulators of inter- and intraspecific competition. However, the extent and mechanisms of how PUA influence diatom–bacteria interactions remain unresolved. In this study, impacts on the diversity of the associated bacterial communities (microbiota) of two contrasting *Skeletonema marinoi* strains (a PUA and a non-PUA producer) were investigated under three nitrate conditions in batch culture. Further, the response of the culture microbiota was studied when spiked with PUA at ecologically relevant concentrations (86nM octadienal and 290nM heptadienal). Of the 741 identified OTUs, Proteobacteria was the most abundant phylum (62.10%), followed by Bacteroidetes (12.33%) and Firmicutes (6.11%). *Escherichia/Shigella* were the most abundant genera for all treatments. Similar communities were present in both spiked and non-spiked cultures suggesting they can tolerate PUA exposure at realistic concentrations. This study suggests that PUA are not major drivers of diatom–bacteria interactions in laboratory cultures.

## 1. Introduction

Diatoms are responsible for approximately 50% of marine primary production [1]. The phycosphere (the organic-matter-rich region surrounding the diatom cell) is host to diverse microbial communities that thrive in close proximity to the diatom, either attached or free living [2]. There is mounting evidence that many diatom species rely on mutualistic interactions with their microbiota [3,4,5,6]. How this balance is maintained is open to conjecture [7], although chemical interactions are considered vital [6,8,9].

The main chemical route is the release by diatoms of dissolved organic matter (DOM) and extracellular metabolites [10,11,12,13], including volatile and cytotoxic polyunsaturated aldehydes (PUA) [3]. PUA, including heptadienal and octadienal, belong to the oxylipin family (products of fatty acids oxidation) [14]. Their broad bioactivities, based on the Michael reaction [15], confer PUA with fairly non-specific toxic properties [16]. PUA are thought to function as part of a multifaceted infochemical approach to bloom regulation through the bottom-up control of herbivore populations [17,18,19,20] and by exerting allelopathic influence over inter- and intraspecifics [21,22,23]. Published data on whether PUA are mediators of diatom–bacteria interactions within the phycosphere are equivocal, but indicate bacteriostatic properties by directly accumulating within their membranes [24], perhaps in response to infection [25]. The bacteriostatic hypothesis argues that PUA act as chemical agents that interfere with bacterial growth and metabolism [26], potentially shifting community structures by changing overall biodiversity [27,28]. However, the bacteriostatic hypothesis is not fully accepted. Indeed, there is some confusion between bacteriostatic and bactericidal abilities [26], with many studies only reporting the cytotoxicity of PUA towards bacteria [3,29].

Nitrogen is the main limiting nutrient for primary productivity in coastal waters [30,31], with concentration variations relative to phosphorous altering the Redfield ratio and changing microalgae growth state and physiology [30]. PUA biosynthesis increases with cell/culture age and under nitrogen limitation [32,33]. Nutrient stress substantially increases the total diatom lipid pool, thereby increasing the quantity of substrate available for PUA synthesis [14]. Diatoms can release PUA from intact and wounded cells, evidenced by *Skeletonema marinoi* releasing heptadienal and octadienal during late stationary growth [34], potentially influencing the culture microbiota.

With renewed interest in elucidating the role of PUA at sea, particularly over oceanic scales [20,27,35,36,37,38,39], it is timely to revisit the bacteriostatic hypothesis, albeit at a laboratory scale. We used next-generation sequencing to address the biodiversity element of the bacteriostatic definition. We aimed to determine whether the bacterial communities associated with cultures of PUA-producing and non PUA-producing strains of *S. marinoi* changed from active (incorporating exponential and linear growth) to late stationary growth phases. Additionally, the effects on the microbiota due to alterations in the Redfield ratio (by varying nitrogen availability) and the deliberate inoculation of PUA at environmentally realistic levels were investigated. Bacterial growth and metabolic function were not investigated.

## 2. Materials and Methods

### 2.1. Experimental Treatments

Two strains of *Skeletonema marinoi*—a PUA producer (SZNFE6) and non-PUA producer (Seasalter, UK) (data not shown)—were cultivated in f/2 medium with added silicate [40] for 22 days. Sterile 250 mL Erlenmeyer flasks containing autoclaved f/2 were inoculated with 5 × 10^6^ cells and sealed with cotton wool and aluminium foil (n = 6 per treatment, n = 3 for media only control). To study the impact of nitrogen availability, the NaNO_3_ concentration was adjusted to three levels: standard f/2 (8.82 × 10^−4^ M), half f/2 (4.41 × 10^−4^ M) or double f/2 (17.64 × 10^−4^ M) (Table S1). To study the effects of PUA addition during active growth (includes both exponential and linear growth phases) and early stationary growth, reference natural secreted PUA concentrations (not PUA potential concentrations) from *S. marinoi* were taken from Vidoudez and Pohnert [34]: 290 nM heptadienal and 86nM octadienal. These concentrations were spiked into the PUA-producing and non-PUA-producing *S. marinoi* cultures to create PUA-spiked treatments from 2E,4E-heptadienal and 2E,4E-octadienal, for each nitrate treatment (Table S1). PUA spiking was repeated twice weekly, allowing for a three-day PUA oxidation period [41]. This method allowed a relatively constant PUA dose to be maintained.

### 2.2. Algal Culture

Cultures were maintained at 19 ± 1 °C with a fixed 16 h light:8 h dark photoperiod with a mean light intensity of 5022 lux using mixed warm and cold fluorescent tubes. The position of the flasks was rotated relative to the light tubes and each flask was manually swirled every second day. Cell counts were made using an improved Neubauer haemocytometer (Hirschmann Laborgeräte GmbH & Co., Eberstadt, Germany) and an Olympus BH-2 microscope (Olympus life science, Waltham, MA, USA) from 50 µL samples.

### 2.3. Nitrate Determination

A rapid spectrophotometric method was used to determine nitrate concentrations [42]. A NaNO_3_ calibration curve was established using serial dilutions with f/2. Two millilitre samples of culture were centrifuged at 3000 *g* for five minutes and the supernatant was analysed at 220 nm in a Cary 100 UV/Vis spectrophotometer (Agilent Technologies, Santa Clara, CA, United States) using a quartz cuvette. To increase accuracy, samples not falling between 0.4 and 1.1 Abs at 220 nm were diluted with f/2 without NaNO_3_ until they fell within the required range. The absorbance values were converted into nitrate concentrations using the linear equation of the calibration curve, y = 2383.4*x* + 0.1183, where y equals the absorbance at 210 nm and *x* equals the concentration of NaNO_3._ The concentrations of the diluted samples were multiplied by their dilution factors.

### 2.4. DNA Extraction

Duplicate 10 mL samples were taken from each flask during the active growth and late stationary phases and frozen at −80 °C until DNA extraction. A PowerSoil^®^ DNA Isolation Kit (QIAGEN, Hilden, Germany) was used, following the manufacturer’s instructions. The culture samples were defrosted, split into 2 mL Eppendorf tubes and centrifuged at 3000 *g* for five minutes, forming a pellet of algae and bacteria cells. The supernatant was extracted and removed. The liquid from the PowerBead tubes was pipetted into the Eppendorf tubes, mixed with the pellet and transferred back into the PowerBead tubes to begin the protocol steps. Nuclease-free water was used to elute the samples, instead of C6 solution due to concerns about EDTA presence. DNA elution from the spin filter membrane was repeated as double eluting the samples increased the yield of DNA. The extracted DNA was stored at −80 °C until sequencing.

### 2.5. Sequence Analysis

Libraries were prepared at NU-OMICS, Northumbria University, with primers targeting the V4 hypervariable region of the 16S rRNA gene following the Schloss wetlab protocol [43]. The library was then sequenced using a V2 500 cycle cartridge on the MiSeq system. Paired end sequences were screened for ambiguous base calls and reads that did not assemble correctly, by removing any reads longer than 275 bp. Forward and reverse sequences were merged and the replicates or low-quality sequences were removed for quality control. All sequences were subsampled to the minimum number of sequences in the 90 samples (755) and due to low read number genetic distance was calculated at 95% allowing the sequences to be clustered into operational taxonomic units (OTUs) (1017). Taxonomic assignment was carried out using Silva.nr_v128 in Mothur to align sequences to the genus level if possible. To ensure that everything overlapped the same region, sequences were screened again for start and end position alignment. Chimera detection was completed using a de novo method in UCHIME [44] through Mothur (version 1.35.1) [45], which removes the chimeric sequences, whereas rare OTUs and sequences identified as non-chimeras were kept. Singleton OTUs were not removed. Negative control samples were used as a proxy for contamination from standards and counts found within negative controls were removed from further analysis, along with unclassified bacteria (245) and archaea (31).

### 2.6. Alpha and Beta Diversity Analysis

Alpha diversity measures were calculated using Mothur to determine the mean species diversity within culture treatments. Richness estimators included Sobs (total number of OTUs per sample), Chao1 (number of rare OTUs), Shannon diversity index (species diversity), Shannon evenness (the numerical closeness of each OTU in an environment) and Inverse Simpson (community richness). Using RStudio (version 1.1.423) and R code for ecological data analysis [46], mean diversity indices were compared for significance. Data did not conform to a normal distribution (*p* < 0.05), therefore the non-parametric Kruskal–Wallis test was carried out, followed by the pairwise Wilcox test with *p* values adjusted using the Benjamini–Hochberg method.

Beta diversity analysis was carried out on phylum, class and genus data, using RStudio and R code for ecological data analysis [46]. The Bray–Curtis dissimilarity index was used to compare the dissimilarities between culture populations and non-metric multi-dimensional scaling (NMDS) allowed for the visual clustering of communities, in terms of grouping factors, and determined any significant differences.

## 3. Results

### 3.1. Culture Growth

**Figure 1:**
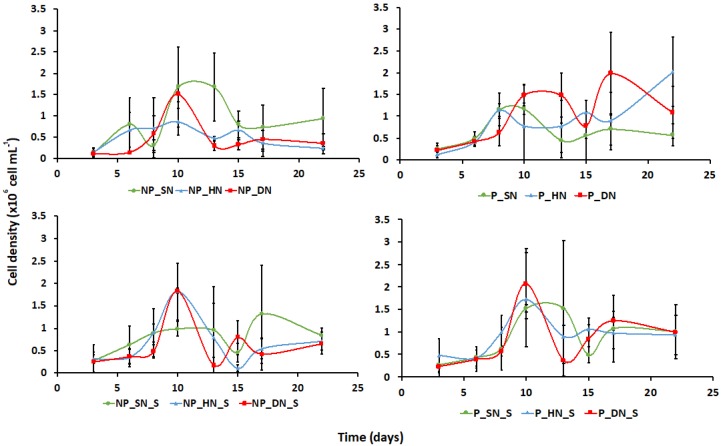
Mean (± standard deviation, n = 3) cell density of 12 *Skeletonema marinoi* treatments over 22 days in 250 mL cultures. Sample key: NP = Non-PUA-producing *S. marinoi*; P = PUA-producing *S. marinoi*; SN = Standard nitrogen relative to f/2 medium (8.82 × 10^−4^ M); HN = Halved nitrogen (4.41 × 10^−4^ M); DN = Double nitrogen (17.64 × 10^−4^ M); S = PUA spiked.

There were no significant differences in culture growth across treatments (Figure 1), with all treatments exhibiting short oscillating stationary phases, particularly in the PUA-producing strains with half nitrate. The large error bars stem from the low (n = 3) replicate numbers.

### 3.2. Sequencing Analysis

The read length for all varied between 253 and 275 base pairs, with a mean read length of 253 (NCBI SRA accession: PRJNA588033). Of the reads, 99.977% classified as bacteria, with the remaining classified as archaea, generating 741 OTUs from 27 phyla, 38 classes, 74 orders, 137 families and 215 genera, excluding unclassified OTUs. A relatively high number of OTUs were unclassified at the genus level, with the non-PUA producing strain containing the highest relative abundance at 13.07% and the controls having the lowest at 6.97% (Table S2).

### 3.3. Community Richness and Diversity Indices

**Figure 2:**
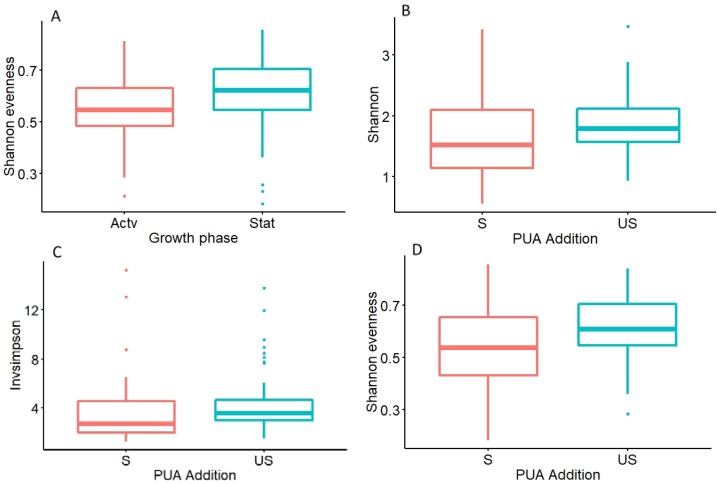
Means (± standard deviation, n = 36) of the alpha diversity measures with significant differences between bacteria communities from *Skeletonema marinoi* cultures. (**A**) Shannon evenness index between active (Actv) and stationary (Stat) growth phases. (**B**) Shannon diversity index between PUA spiked (S) and unspiked (US) treatments. (**C**) Inverse Simpson index (Invsimpson) between PUA spiked (S) and unspiked (US) treatments. (**D**) Shannon evenness index between PUA spiked (S) and unspiked (US) treatments.

Four treatments had statistically significant alpha diversity indices (Figure 2); Shannon evenness and growth phase (active median = 34.2 ± 18.4, stationary median = 31.0 ± 28.0) (Kruskal–Wallis K = 5.4577, df = 1, *p* < 0.05), InvSimpson and PUA addition (spiked median = 20.7 ± 9.48, unspiked median = 20.9 ± 12.3) (Kruskal–Wallis K = 6.5609, df = 1, *p* < 0.05), Shannon evenness and PUA addition (spiked median = 0.536 ± 0.223, unspiked median = 0.608 ± 0.160) (Kruskal–Wallis K = 5.7837, df = 1, *p* < 0.05) and Shannon and PUA addition (spiked median = 1.52 ± 0.959, unspiked median = 1.79 ± 0.549) (Kruskal–Wallis K = 5.0186, df = 1, *p* < 0.05).

Communities were sampled at 0.05 genetic distance and subsampled at 755 sequences; however, to capture the range of diversity present, rarefaction curves were created using the complete number of sequences for each sample (Figures S1 and S2). Rarefaction curves for the control treatment were close to saturation. The non-PUA- and PUA-producing strains were close to saturation for the stationary phase, whereas both strains in the active phase were far from saturation.

### 3.4. Bacterial Community Composition

The number of unclassified bacterial sequences from the controls were much lower than for the two *S. marinoi* strains; this along with the saturated rarefaction curve suggests that the community was accurately characterised. From the rarefaction analysis and high number of unclassified sequences of the two strains, microbial diversity could be higher than observed, with a possibility of novel bacteria within the phycosphere. Control and strains contained similar genera.

**Figure 3:**
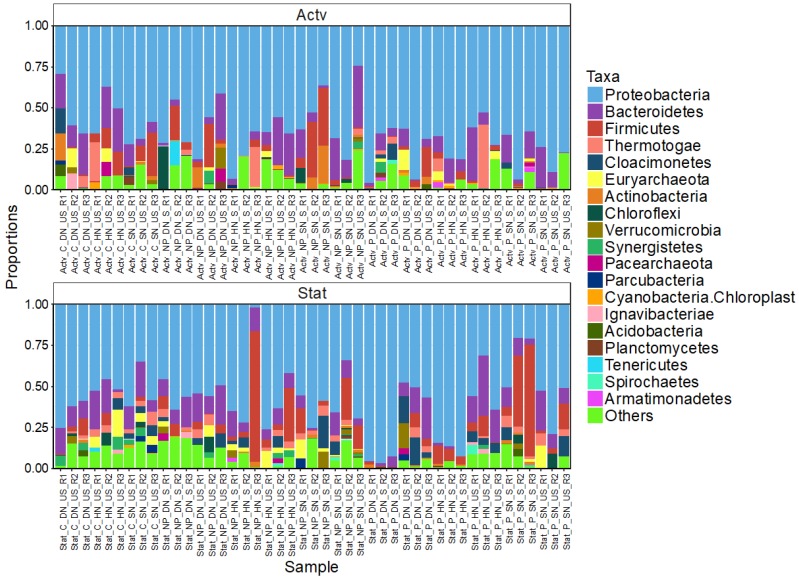
The relative abundance of bacteria, classified at the phylum level, from different cultured treatments of *Skeletonema marinoi.* Sample key: Actv = Active growth phase; Stat = Stationary growth phase; NP = Non-PUA-producing strain; P = PUA-producing strain; SN = Standard nitrogen relative to f/2 medium (8.82 × 10^−4^ M); HN = Halved nitrogen (4.41 × 10^−4^ M); DN = Double nitrogen (17.64 × 10^−4^ M); S = PUA spiked; US = Unspiked; R = Replicate treatment. “Other” included the 15 least abundant phyla.

**Figure 4:**
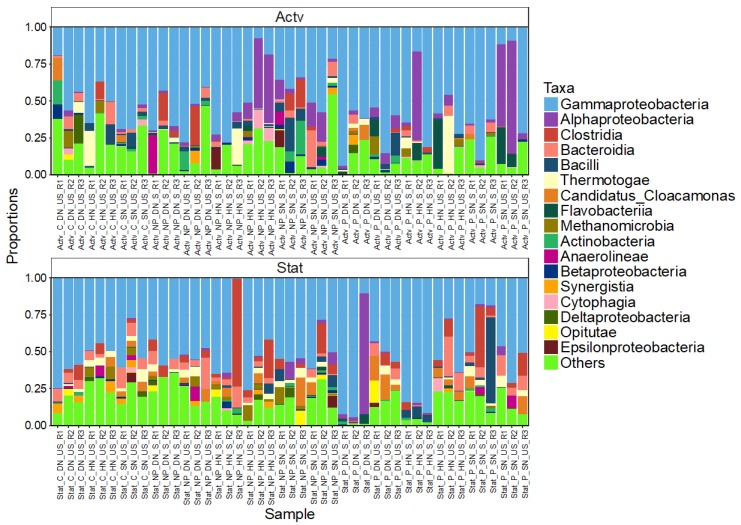
The relative abundance of bacteria, classified at the class level, from different cultured treatments of *Skeletonema marinoi.* Sample key: Actv = Active growth phase; Stat = Stationary growth phase; NP = Non-PUA-producing strain; P = PUA-producing strain; SN = Standard nitrogen relative to f/2 medium (8.82 × 10^−4^ M); HN = Halved nitrogen (4.41 × 10^−4^ M); DN = Double nitrogen (17.64 × 10^−4^ M); S = PUA spiked; US = Unspiked; R = Replicate treatment. “Other” included the 25 least abundant classes.

The relative abundance of phylum level OTUs (Figure 3) indicated that certain OTUs were present in all samples whereas others were treatment specific. The most abundant phyla across all treatments were the Proteobacteria (62.10%), Bacteroidetes (12.33%) and Firmicutes (6.11%). At class level Gammaproteobacteria were the most abundant, followed by Alphaproteobacteria (Figure 4). The most common genus was *Escherichia/Shigella*, from the order Enterobacteriales, present 33,482 times across all samples, followed by *Enterococcus* (class Bacilli, order Lactobacillates) and *Pseudomonas* (class Gammaproteobacteria, order Pseudomonadales) (Figure S3).

**Figure 5:**
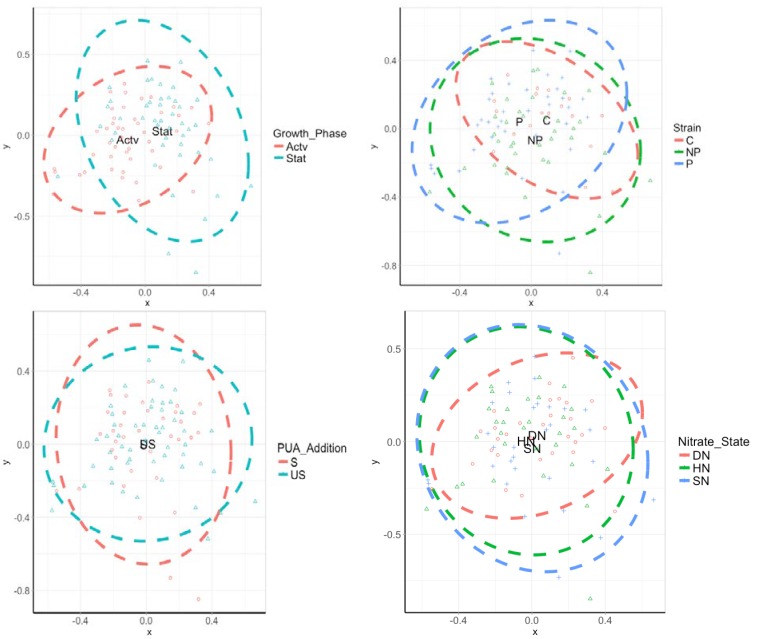
Two-dimensional non-metric multi-dimensional scaling plot (NMDS) of a Bray–Curtis dissimilarity matrix for OTU-level taxonomic data. Each point symbolises a cultured sample. Clustered points show high similarity. Dashed lines show 95% confident limits and the letters show the centre of the community. Sample key: Actv = Active growth phase; Stat = Stationary growth phase; NP = Non-PUA-producing strain; P = PUA-producing strain; SN = Standard nitrogen relative to f/2 medium (8.82 × 10^−4^ M); HN = Halved nitrogen (4.41 × 10^−4^ M); DN = Double nitrogen (17.64 × 10^−4^ M); S = PUA spiked; US = Unspiked.

Treatment-associated OTUs all overlapped at the phylum level, with only loose clustering by treatment. Community clusters had slight separation by growth phase and PUA additions; however, there was no evidence of separation between nitrate concentrations or shifts in communities between the *S. marinoi* strains (Figure S4). At the class level, the 95% confident limits were similar for all treatments within strain and nitrate state, whereas the active and stationary phases had less overlap (Figure S5). Spiked and unspiked cultures had similar communities at the genus level, with most samples overlaying. In terms of nitrate state, double nitrate had the tightest 95% confident limits although high clustering from all three states was apparent. The active and stationary growth phases had the least similar communities, while the treatment strain had different 95% confidence limits (Figure S6). At OTU level, the active and stationary growth phases had the most distant communities of all the taxonomic levels analysed and of all factors at this level (Figure 5). The addition of artificial PUAs and changing nitrate conditions did not separate the communities. Differences between the controls and two strains had only a slight effect on the clusters.

There were significant differences in assemblage dissimilarity between growth phases, strains, nitrate states and PUA additions for three phyla (Proteobacteria, Actinobacteria and Chloroflexi), with the former significantly different across all four treatments, whereas SR1 had no significant differences in assemblage dissimilarity between the treatments (Tables S3–S6). The Gammaproteobacteria were more influenced by overall trends in nitrate availability, while Bacteroidia and unclassified OTUs featured more heavily in the stationary growth phase compared to the active growth phase. For the spiked treatments, Methanomicrobia were associated with unspiked cultures whereas Alphaproteobacteria were more strongly associated with the PUA-producing strain. Under higher resolution examination, significant differences at the genus level were apparent, with *Marinobacter* and *Phenylobacterium* associated with the active phase and *Thermovirga* with the stationary phase. *Pseudomonas* and *Methanothrix* were associated with the nitrate deplete treatments. *Marivita*, *Hoeflea*, *Oleiphilus*, *Maricaulis* and *Haliea* were more commonly associated with the PUA-producing strain.

## 4. Discussion

Exposure to PUA reportedly induces varying effects on marine bacteria (bactericidal and bacteriostatic). We further addressed this issue by questioning whether the microbiota associated with contrasting growth phases of laboratory cultures of PUA-producing and non-producing strains of *Skeletonema marinoi* differed with respect to nitrate state and following a pattern of deliberate and sustained inoculation with exogenous PUA at environmentally relevant concentrations.

In both natural and culture environments, diatoms and bacteria live in symbiotic and often mutualistic associations, generally mediated by the exchange of extracellular metabolites [5]. In this study, the diatom cell density increased as expected in the active growth phase (encompassing exponential and linear patterns characteristic of batch cultivation) when nutrients were replete; however, the stationary phase was characterised by oscillating cell densities, again indicative of batch culture, evidencing short-lived cycles of nutrient remineralisation (likely bacteria mediated) and renewed uptake by the diatoms [47]. This internal recycling of nutrient pools is common in batch systems where no other factors are limiting, whereupon the cultures will enter senescence and achieve peak PUA release [34]. Repeated spiking with octadienal and heptadienal at environmentally relevant concentrations was undertaken to simulate grazing and infection events [48]. The alpha diversity indices were significantly different between spiked and unspiked treatments highlighting that PUA do influence bacterial diversity [49]; however, the community composition overlapped with only slight separation in core communities, and no separation at the genus level, indicating that PUA effects, although present, were minor.

According to Riemann et al. [50], the post bloom phase is accompanied by alterations in bacterial community composition, suggesting that community shifts do occur, with multiple studies showing that phytoplankton succession is one of the most important factors affecting bacterial community composition and abundance [51,52]. The underlying mechanism is proposed to relate to the gradual release of phytoplankton extracellular products [52]. With respect to PUA-producing diatoms, a PUA concentration gradient is established within the phycosphere [49], particularly during the late stationary and senescent phases, with evidence that the role of PUA concentration is important in regulating bacterial metabolism in sinking phytoplankton particles, i.e., marine snow [28]. This should provide a spatiotemporal vehicle for bacterial succession. However, the lack of significant differences between the two strains during both the active and stationary growth phases suggests that any naturally released PUA were not sufficient to cause any driving effect. As shown by Paul and co-workers [49], the bacterial community composition is growth phase dependent. At all taxonomic levels, the growth phase samples had slightly separated community centres and the most distant 95% confident limits out of all treatments, although the overall effect on diatom–bacteria interactions was limited.

Nitrogen is a limiting nutrient for microalgae growth in coastal waters. Nitrogen additions may affect PUA production in diatom blooms [32], exacerbating toxic effects on grazing copepods [53]. Natural PUA production decreases under nitrate-limited conditions due to limited enzyme activity [23]. However, concentrations of total particulate PUA do not positively correlate with nutrient conditions [54]. Nutrient limitation can also significantly increase the amount of extracellular products released by diatoms, including PUA, and nutritional constraints at the end of a bloom increases PUA release [3,16]. Our data suggests that nitrate does not limit or significantly alter microbiota composition, corroborating previous studies [50,55]; however, significant correlations between species richness and nutrient availability during increased nutrient supply have also been documented [56].

Nutrient stress can influence the bioavailability of dissolved organic matter (DOM) for both diatoms and bacteria [57]. Whilst not directly investigated, the wider effects in a natural situation are important to consider. Alterations in organic matter during a bloom can change the dominant bacteria species to those with more suitable metabolic capabilities [58], although not in all instances [59]. Furthermore, Logue et al. [60] found a significant relationship for Alphaproteobacteria due to the growth of bacteria on phytoplankton-derived DOM, leading to increased growth and enzymatic activities [58].

Microbial communities are generally dominated by a small subset of highly abundant taxa. The numerically uncommon but metabolically more diverse taxa are often overlooked yet are ordinarily fundamental to community dynamics and the maintenance of a stable and functional microbiome [56]. The relatively slow centrifugation force used in our study (3000 *g*) will have likely disproportionately sampled the larger bacteria and those attached to the diatoms. The consequence of which is an underrepresentation of small bacteria from our community analysis. It would be interesting to repeat this work with a higher spin speed to get a more comprehensive view of community dynamics. The microbial communities—dominated by the Proteobacteria, Bacteriodetes, Gammaproteobacteria and *Escherichia/Shigella*, also found in previous studies [24,58,61,62]—did change with growth phase and in response to PUA spiking, albeit at a relatively subtle level. Curiously, despite different *Skeletonema* species, including *S. marinoi*, having antimicrobial capacities [63,64,65], the strains of *S. marinoi* used had little to no effect on the microbiota, nor did the nitrate treatments. Although, the microbial communities present in both *S. marinoi* strains did not differ significantly, the non-PUA producing strain had more OTUs and a greater abundance of rare OTUs. This indicates a high percentage of naturally occurring bacteria are resistant to PUA as suggested by Paul and co-workers [49], or that any negative effects caused by PUA for some taxa were buffered by positive effects supported by the diversity of the community, i.e., a degree of functional redundancy [66].

The abundance of Gammaproteobacteria increases as blooms progress whereas Alphaproteobacteria decrease [4], evidently related to their abilities to degrade organic matter [67,68]. However, *Marinobacter* differed between growth phases, being associated with active growth. Gammaproteobacteria, *Haliea* in particular, increased in abundance with the PUA-producing strain, demonstrating a capacity to thrive in the presence of PUA. This PUA tolerance suggests either some form of compensatory mechanism among species lowers their sensitivity, or highly resistant taxa are present within the community [66]. Two of the most abundant Gammaproteobacteria genera (*Escherichia/Shigella* and *Pseudomonas*) dominated throughout all culture conditions. Balestra et al.’s results contradict these findings as Gammaproteobacteria abundance remained unaffected to exposure of 2E,4E/Z-decadienal; however, they did experience reduced metabolic activities, particularly when a PUA mixture was added (indicative of synergistic interactions). These differences may have arisen from Balestra et al. using natural bacterial populations versus our laboratory-adapted communities. They also used lower PUA concentrations (7.5 nM of heptadienal and octadienal in combination versus our 290 and 86 nM, respectively) over a shorter timescale (6–24 h versus our 22 days) [66].

Gammaproteobacteria abundance also increases in natural blooms particularly in nutrient rich waters [62,69], analogous to the double nitrate f/2 treatment. However, the association of *Pseudomonas* with nitrate deplete treatments is contrary to this preference as *Pseudomonas* has been reported to use nitrate as an alternative electron acceptor, allowing anaerobic growth [70]. Gammaproteobacteria have many important ecological functions, including carbon fixation in the Calvin–Benson–Bassham cycle during diatom blooms [71] and anaerobic metabolism in the sulphur reduction II pathway allowing diatoms to survive in dark, anoxic conditions [71,72]. An intriguing offshoot of this study would be to define the microbiota associated with diatom resting stages to determine whether the microbiota and/or the sediment-associated communities have a role in sustaining the cysts, particularly over extended periods of sediment cover [73,74].

In PUA-producing treatments the Alphaproteobacteria showed low relative abundances during the stationary phase despite having been reported as having high PUA resistance [66], whereas Bacteroidia abundance increased, suggesting differential sensitivity to PUAs. Bacteroidetes and Firmicutes were relatively unaffected by PUA addition, possibly due to their specialised role in degrading phytoplankton DOM [68]. This is further supported by select species being able to degrade PUA into smaller hydrocarbons as an energy source—a potential adaption for PUA-rich environments [24]. Furthermore, Firmicutes can dominate highly toxic blooms, increasing their abundance with bloom succession [4].

Rhodobacterales had low abundances in most cultures, suggesting some PUA sensitivity; however, they may develop resistance over time when natural PUA concentrations are steadily released [66]. *Marivita* was associated with the PUA-producing strain, which is more fitting with the literature. This free-living bacteria can dominate in diatom cultures [51] by following the same growth progression as the diatoms, benefitting from released DOM [69].

This study reveals subtle interplays within diatom culture microbiota, influenced by culture age and exogenous PUA availability, although not by diatom strain or nitrogen condition, and furthers the limited knowledge about PUA-derived interactions. Nevertheless, the likelihood that this may be an artefact of our cultivation practice must be considered. With common seawater sources and growth conditions, the respective culture bacterial communities may have become homogeneous over the extended periods these strains have been grown. Equally, our experimental approach is not representative of the enormous genetic diversity of diatoms and their associated microflora in nature. Therefore, we cannot discount the possibility that fresh isolates and in situ phenotypes would host divergent microbial communities in direct response to their immediate biotic and abiotic selective pressures [28,49]. Equally, sufficient nitrate would still have been present in the half-f/2 treatment to avoid, or at least ameliorate, nitrate stress. A stricter state of nitrate deprivation, perhaps simulating stratified surface water levels, may have elicited a more definitive community response.

Many bacterial groups could tolerate PUA presence, an important competitive advantage in a natural bloom. Given the small shifts in community diversity and composition, PUA production is unlikely to be a major driver of diatom–bacteria interactions, at least in well-established laboratory scale cultures, but may play a role as part of a more diverse bouquet of diatom chemical signals.

## Supplementary Materials

The following are available online at https://www.mdpi.com/2075-1729/10/3/29/s1. Figure S1: Rarefaction curves for control samples taken during the active growth phase. Figure S2: Rarefaction curves for control samples taken during the stationary growth phase. Figure S3: The relative abundance of bacteria, classified at genus level, from different cultured treatments of *Skeletonema marinoi.* Figure S4: Two-dimensional non-metric multi-dimensional scaling plot on Bray–Curtis dissimilarity matrix for phylum level taxonomic data. Figure S5: Two-dimensional non-metric multi-dimensional scaling plot on Bray–Curtis dissimilarity matrix for class level taxonomic data. Figure S6: Two-dimensional non-metric multi-dimensional scaling plot on Bray–Curtis dissimilarity matrix for genus level taxonomic data. Table S1: Experimental design of the cultured flasks. Table S2: The relative abundance (%) of unclassified sequences. Table S3: Bray–Curtis dissimilarity r^2^ and *p* values between active and stationary growth phases. Table S4: Bray–Curtis dissimilarity r^2^ and *p* values for between the PUA producer strain, non-PUA producer strain and controls. Table S5: Bray–Curtis dissimilarity r^2^ and *p* values for between three nitrate states. Table S6: Bray–Curtis dissimilarity r^2^ and *p* values for between spiked *Skeletonema marinoi* cultures with aldehydes.
