# Supplementary material for: Diatom-Derived Polyunsaturated Aldehydes Are Unlikely to Influence the Microbiota Composition of Laboratory-Cultured Diatoms
Source: Life (Basel). 2020 Mar 24;10(3):29. doi: 10.3390/life10030029 (PMC7151586; doi:10.3390/life10030029)
Supplement: Supplementary file 1 [file life-10-00029-s001.zip › life-705266-SI.docx]

Supplementary Materials for Diatom-Derived Polyunsaturated Aldehydes are Unlikely to Influence the Microbiota Composition of Laboratory-Cultured Diatoms

Chloe L. Eastabrook ^1^, Paul Whitworth ^1^_,_ Georgina Robinson ^2^ and Gary S. Caldwell ^1,^*

^1^ School of Natural and Environmental Sciences, Newcastle University, Newcastle upon Tyne, NE1 7RU, UK; c.l.eastabrook1@ncl.ac.uk (C.L.E.); p.whitworth@ncl.ac.uk (P.W.)

^2^ Scottish Association for Marine Science, Scottish Marine Institute, Oban, PA37 1QA, UK; Georgina.Robinson@sams.ac.uk

***** Correspondence: gary.caldwell@ncl.ac.uk; Tel.: +44-191-208-6660

**Table S1.** Experimental design of the cultured flasks. Controls contained f/2 medium only. Standard nitrogen relative to f/2 medium (8.82 × 10^−4^ M), halved nitrogen (4.41 × 10^−4^ M) and double nitrogen (17.64 × 10^−4^ M). Spiked with aldehydes 2E,4E-heptadienal and 2E,4E-octadienal. Each row was produced in triplicate.

| **Strain** | | | **Nitrate Condition** | | | **PUA Addition** | |
| --- | --- | --- | --- | --- | --- | --- | --- |
| **PUA** | **Non-PUA** | **Control** | **Half f/2** | **f/2** | **Double f/2** | **Spiked** | **Unspiked** |
| + |  |  | + |  |  | + |  |
| + |  |  |  | + |  | + |  |
| + |  |  |  |  | + | + |  |
| + |  |  | + |  |  |  | + |
| + |  |  |  | + |  |  | + |
| + |  |  |  |  | + |  | + |
|  | + |  | + |  |  | + |  |
|  | + |  |  | + |  | + |  |
|  | + |  |  |  | + | + |  |
|  | + |  | + |  |  |  | + |
|  | + |  |  | + |  |  | + |
|  | + |  |  |  | + |  | + |
|  |  | + | + |  |  |  |  |
|  |  | + |  | + |  |  |  |
|  |  | + |  |  | + |  |  |

**Figure S1.** Rarefaction curves for control samples (**A**), non-PUA producing *Skeletonema marinoi* (**B**) and PUA producing *S. marinoi* (**C**) taken during the active growth phase.

**Figure S2.** Rarefaction curves for control samples (**A**), non-PUA producing *Skeletonema marinoi* (**B**) and PUA producing *S. marinoi* (**C**) taken during the stationary growth phase.

**Table S2.** The relative abundance (%) of unclassified sequences at five taxonomic levels for 90 DNA samples taken from cultures of *Skeletonema marinoi*.

| **Taxonomy** | **Non-PUA strain** | **PUA strain** | **Controls** |
| --- | --- | --- | --- |
| Phylum | 3.16 | 2.01 | 1.55 |
| Class | 5.51 | 3.78 | 3.73 |
| Order | 6.89 | 5.28 | 4.35 |
| Family | 8.78 | 7.28 | 5.72 |
| Genus | 13.07 | 11.37 | 6.97 |

**Figure S3.** The relative abundance of bacteria, classified at genus level, from different cultured treatments of *Skeletonema marinoi.* Sample key: Actv = Active growth phase, Stat = Stationary growth phase, NP = non-PUA producing strain, P = PUA producing strain, SN = Standard nitrogen relative to f/2 medium (8.82 × 10^−4^ M), HN = Halved nitrogen (4.41 × 10^−4^ M), DN = Double nitrogen (17.64 × 10^−4^ M), S = Spiked with aldehydes 2E,4E-heptadienal and 2E,4E-octadienal, US = unspiked, R = replicates. ‘Other’ included the 25 least abundant genera.

**Table S3.** Bray-Curtis dissimilarity r^2^ and P values between active and stationary growth phases of *Skeletonema marinoi* cultures. *** = strong significance, * = weak significance.

| **Phylum** | **r^2^** | **P** | **Strength of Significance** |
| --- | --- | --- | --- |
| Proteobacteria | 0.4314 | 0.001 | *** |
| Bacteroidetes | 0.1297 | 0.005 | ** |
| Actinobacteria | 0.1922 | 0.001 | * |
| Chloroflexi | 0.0239 | 0.356 | *** |
| Verrucomicrobia | 0.16 | 0.002 | ** |
| Synergistetes | 0.1407 | 0.002 | ** |
| SR1 | 0.0297 | 0.282 |  |
| Lentisphaerae | 0.0361 | 0.199 |  |
| Chlamydiae | 0.0263 | 0.343 |  |

**Table S4.** Bray-Curtis dissimilarity r^2^ and P values for between the PUA producer strain, non-PUA producer strain and controls of *Skeletonema marinoi* cultures. *** = strong significance, * = weak significance.

| **Phylum** | **r^2^** | **P** | **Strength of Significance** |
| --- | --- | --- | --- |
| Proteobacteria | 0.4314 | 0.001 | *** |
| Bacteroidetes | 0.1297 | 0.005 | ** |
| Actinobacteria | 0.1922 | 0.001 | *** |
| Chloroflexi | 0.0239 | 0.356 |  |
| Verrucomicrobia | 0.16 | 0.002 | ** |
| Synergistetes | 0.1407 | 0.002 | ** |
| SR1 | 0.0297 | 0.282 |  |
| Lentisphaerae | 0.0361 | 0.199 |  |
| Chlamydiae | 0.0263 | 0.343 |  |

**Table S5.** Bray-Curtis dissimilarity r^2^ and P values for between three nitrate states (standard nitrogen relative to f/2 medium (8.82 × 10^−4^ M), halved nitrogen (4.41 × 10^−4^ M), double nitrogen (17.64 × 10^−4^ M)) of *Skeletonema marinoi* cultures. *** = strong significance, * = weak significance.

| **Phylum.** | **r^2^** | **P** | **Strength of Significance** |
| --- | --- | --- | --- |
| Proteobacteria | 0.4329 | 0.001 | *** |
| Actinobacteria | 0.1848 | 0.001 | *** |
| Chloroflexi | 0.0235 | 0.372 |  |
| Ignavibacteriae | 0.1039 | 0.005 | ** |
| SR1 | 0.0313 | 0.254 |  |
| Latescibacteria | 0.0375 | 0.197 |  |
| Gemmatimonadetes | 0.0547 | 0.1 |  |

**Table S6.** Bray-Curtis dissimilarity r^2^ and P values for between spiked *Skeletonema marinoi* cultures with aldehydes 2E,4E-heptadienal and 2E,4E-octadienal and unspiked cultures. *** = strong significance, * = weak significance.

| **Phylum** | **r^2^** | **P** | **Strength of Significance** |
| --- | --- | --- | --- |
| Proteobacteria | 0.4331 | 0.001 | *** |
| Bacteroidetes | 0.1302 | 0.002 | ** |
| Actinobacteria | 0.1843 | 0.001 | *** |
| Chloroflexi | 0.0235 | 0.332 |  |
| Planctomycetes | 0.0678 | 0.034 | * |
| Tenericutes | 0.0185 | 0.497 |  |
| Armatimonadetes | 0.0512 | 0.1 |  |
| SR1 | 0.0313 | 0.246 |  |


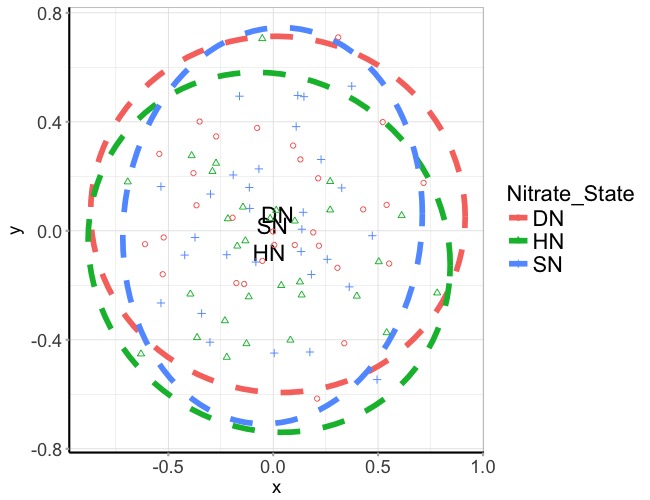

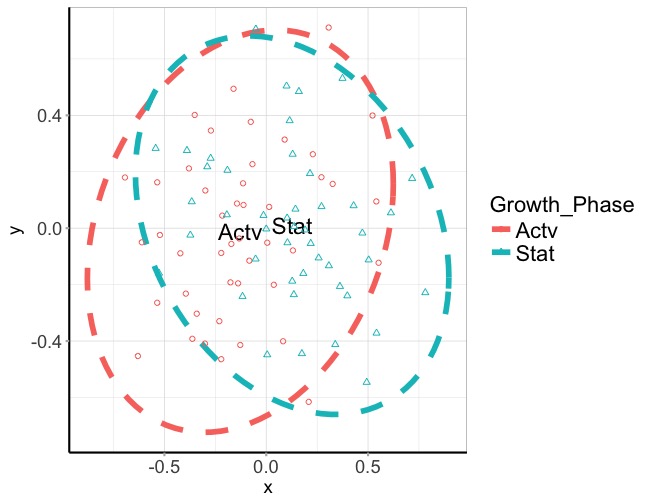

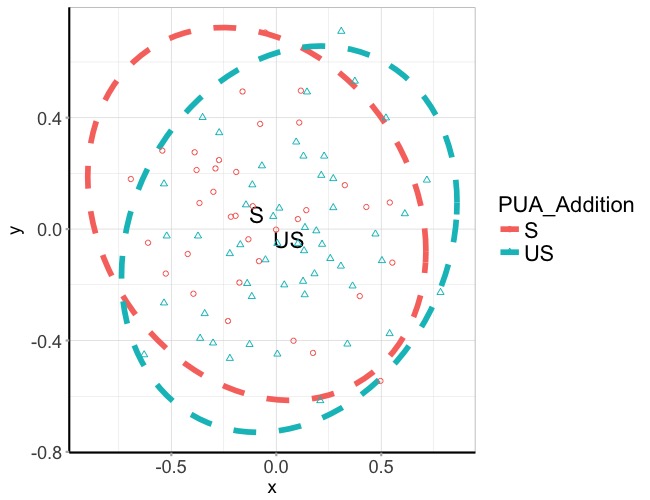

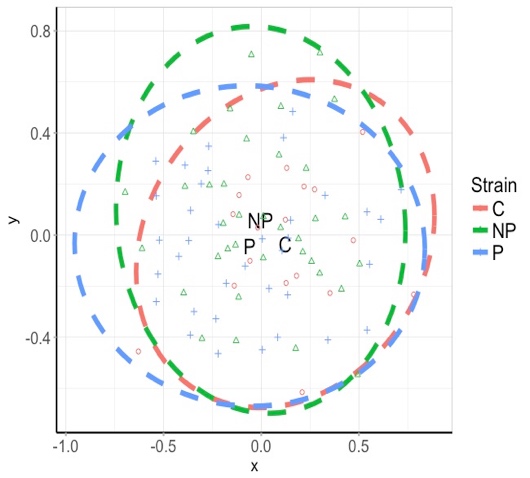


**Figure S4.** Two-dimensional non-metric multi-dimensional scaling (NMDS) plot on Bray-Curtis dissimilarity matrix for phylum level taxonomic data. Each point symbolises a cultured sample. Clustered points show high similarity. Dashed lines show 95% confident limits and the letters show the centre of the community. Sample key: Actv = Active growth phase, Stat = Stationary growth phase, NP = non-PUA producing strain, P = PUA producing strain, SN = Standard nitrogen relative to f/2 medium (8.82 × 10^−4^ M), HN = Halved nitrogen (4.41 × 10^−4^ M), DN = Double nitrogen (17.64 × 10^−4^ M), S = Spiked with aldehydes 2E,4E-heptadienal and 2E,4E-octadienal, US = Unspiked.


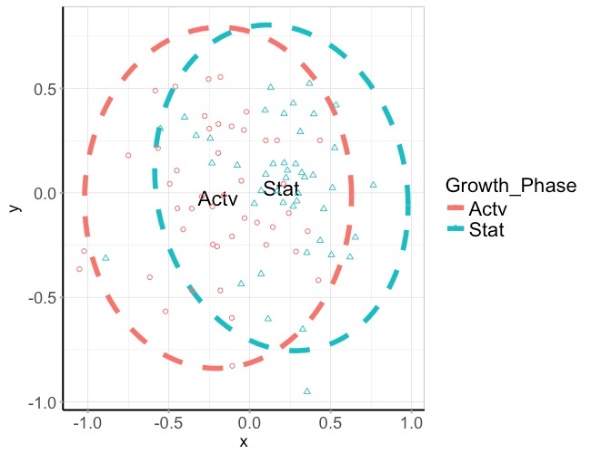

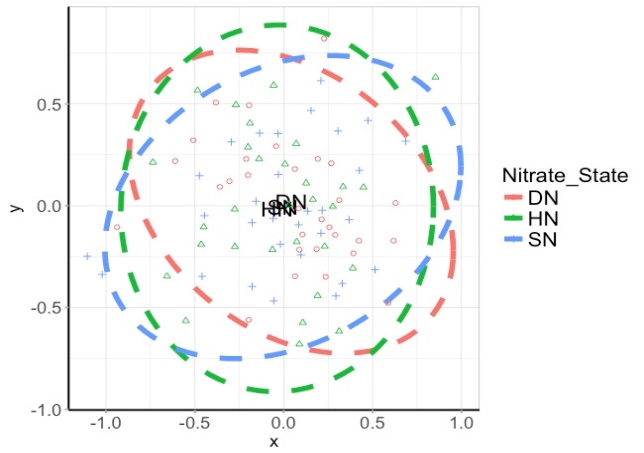

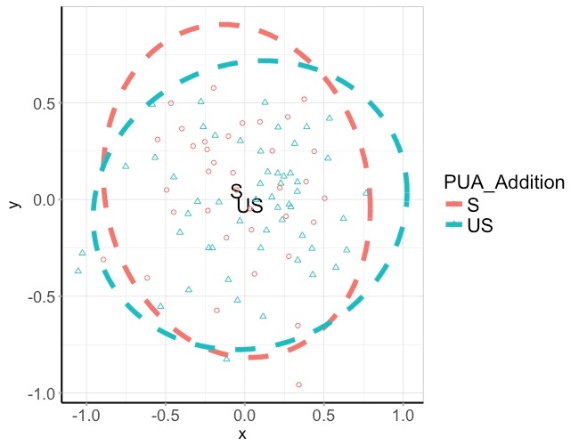

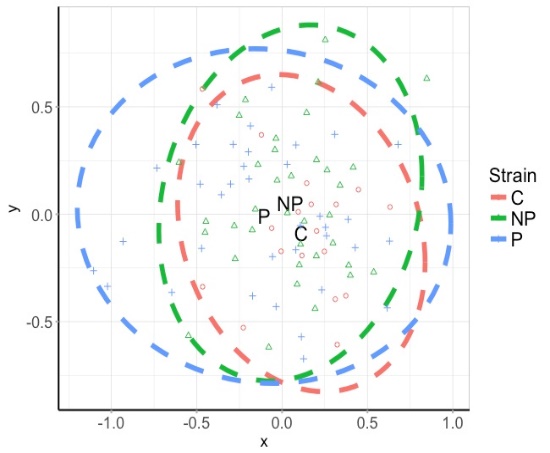


**Figure S5.** Two-dimensional non-metric multi-dimensional scaling plot (NMDS) on Bray-Curtis dissimilarity matrix for class level taxonomic data. Each point symbolises a cultured sample. Clustered points show high similarity. Dashed lines show 95% confident limits and the letters show the centre of the community. Sample key: Actv = Active growth phase, Stat = Stationary growth phase, NP = non-PUA producing strain, P = PUA producing strain, SN = Standard nitrogen relative to f/2 medium (8.82 × 10^−4^ M), HN = Halved nitrogen (4.41 × 10^−4^ M), DN = Double nitrogen (17.64 × 10^−4^ M), S = PUA Spiked, US = Unspiked.


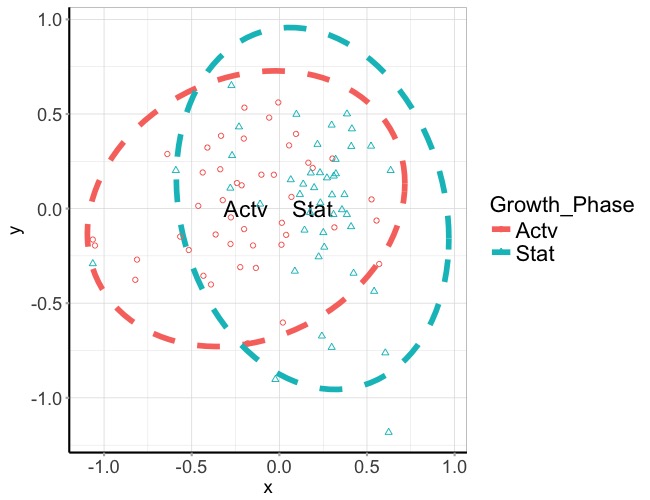

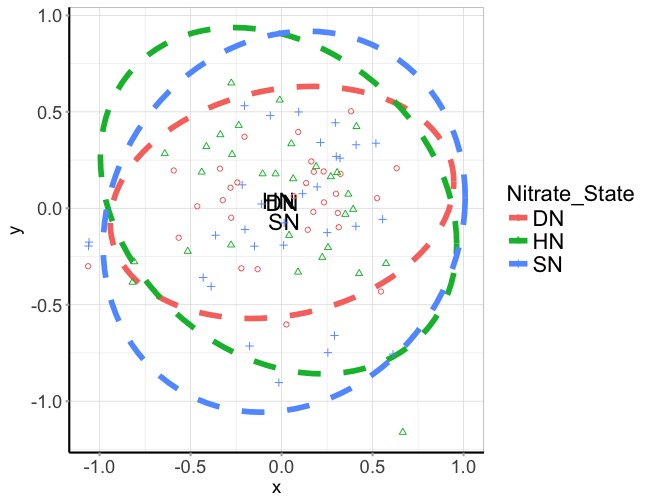

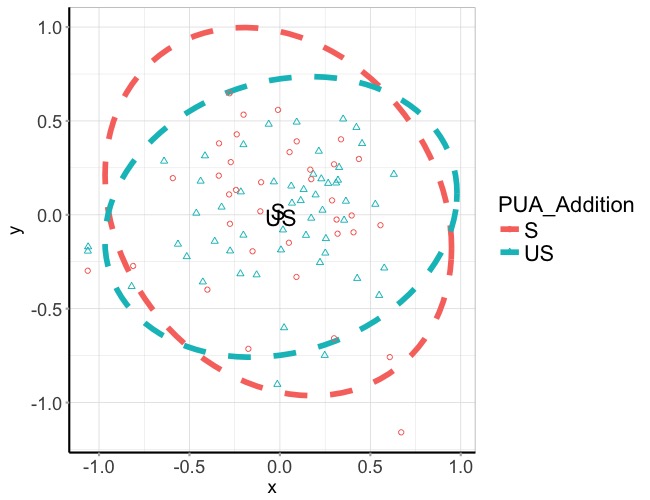

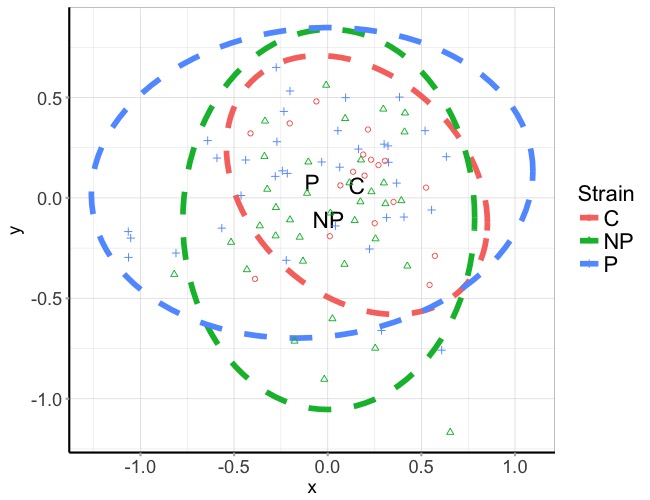


**Figure S6.** Two-dimensional non-metric multi-dimensional scaling (NMDS) plot on Bray-Curtis dissimilarity matrix for genus level taxonomic data. Each point symbolises a cultured sample. Clustered points show high similarity. Dashed lines show 95% confident limits and the letters show the centre of the community. Sample key: Actv = Active growth phase, Stat = Stationary growth phase, NP = non-PUA producing strain, P = PUA producing strain, SN = Standard nitrogen relative to f/2 medium (8.82 × 10^−4^ M), HN = Halved nitrogen (4.41 × 10^−4^ M), DN = Double nitrogen (17.64 × 10^−4^ M), S = Spiked with aldehydes 2E,4E-heptadienal and 2E,4E-octadienal, US = Unspiked.

| 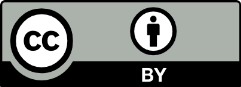 | 1. © 2020 by the author. Licensee MDPI, Basel, Switzerland. This article is an open access 2. article distributed under the terms and conditions of the Creative Commons Attribution   (CC BY) license (http://creativecommons.org/licenses/by/4.0/). |
| --- | --- |
